# Smoking and Lung Cancer: A Geo-Regional Perspective

**DOI:** 10.3389/fonc.2017.00194

**Published:** 2017-09-01

**Authors:** Zahraa Rahal, Shaza El Nemr, Ansam Sinjab, Hassan Chami, Arafat Tfayli, Humam Kadara

**Affiliations:** ^1^Faculty of Arts and Sciences, Department of Biology, American University of Beirut, Beirut, Lebanon; ^2^Faculty of Medicine, Department of Biochemistry and Molecular Genetics, American University of Beirut, Beirut, Lebanon; ^3^Faculty of Medicine, Department of Internal Medicine, American University of Beirut, Beirut, Lebanon; ^4^Department of Epidemiology, Division of Cancer Prevention, University of Texas MD Anderson Cancer Center, Houston, TX, United States

**Keywords:** lung cancer, smoking, pathogenesis, early detection, prevention, global smoking patterns

## Abstract

Lung cancer is the leading cause of cancer-related deaths worldwide. Non-small cell lung cancer (NSCLC) represents the most frequently diagnosed subtype of this morbid malignancy. NSCLC is causally linked to tobacco consumption with more than 500 million smokers worldwide at high risk for this fatal malignancy. We are currently lagging in our knowledge of the early molecular (e.g., genomic) effects of smoking in NSCLC pathogenesis that would constitute ideal markers for early detection. This limitation is further amplified when considering the variable etiologic factors in NSCLC pathogenesis among different regions around the globe. In this review, we present our current knowledge of genomic alterations arising during early stages of smoking-induced lung cancer initiation and progression, including discussing the premalignant airway field of injury induced by smoking. The review also underscores the wider spectra and higher age-adjusted rates of tobacco (e.g., water-pipe smoke) consumption, along with elevated environmental carcinogenic exposures and relatively poorer socioeconomic status, in low-middle income countries (LMICs), with Lebanon as an exemplar. This “cocktail” of carcinogenic exposures warrants the pressing need to understand the complex etiology of lung malignancies developing in LMICs such as Lebanon.

## Smoking Exposure and Lung Cancer

Lung cancer is the most common malignancy worldwide with 1.8 million new cases and ~1.6 million deaths in 2012 (1). Lung cancer consists of two main subtypes, small-cell lung cancer (SCLC) and non-small cell lung cancer (NSCLC), with the latter accounting for approximately 85% of diagnosed lung malignancies (2). The overwhelming majority (~85%) of diagnosed lung cancers develop in lifetime (former or current) smokers (3, 4). Despite recent advances in treatment of lung cancer (e.g., targeted therapy), the overall prognosis for the disease remains dismal with an estimated 5-year survival rate in the US of 18% (5). Of note, prognosis of smoker lung cancer patients is markedly lower than patients who have never smoked (6, 7). These low survival rates are largely due to an advanced stage at diagnosis in the majority of patients (8), and a high relapse rate in patients presenting with early-stage disease (9). This conundrum warrants new strategies for early detection and prevention of lung cancer, which, to date, have been extremely limited.

Consumption of tobacco (mainly by cigarette smoking) is causally related to lung cancer (2, 10). Smoking cessation is an important behavioral measure for lung cancer prevention. Yet, former smokers still exhibit elevated risk compared to never smokers as this risk never returns to baseline (11–13). Indeed, almost 50% of diagnosed lung cancer cases occur in former smokers (14). As such, there are over 500 million smokers worldwide at elevated risk of lung disease including cancer (15). It is noteworthy that only a fraction (~15%) of smokers develop lung tumors in their lifetime (16). We are still unable to identify, with precision, smokers at highest risk for developing this malignancy. Notably, the National Lung Screening Trial demonstrated a 20% reduction in mortality with low-dose CT (LDCT) screening (17), and guidelines now endorse annual LDCT for those at increased risk (18). Yet, high false-positive screen rates, cumulative radiation exposure, and substantial economic costs have been associated with LDCT screening (2, 19).

This review discusses the early molecular pathology of smoking-induced lung cancer. It also explores genomic alterations that have been reported in “normal” (airway field of injury) and premalignant phases of NSCLC pathogenesis as well as in early-stage disease. Of note, the review will also center on the burden of lung cancer and its region-specific epidemiology in low-middle income countries (LMICs; e.g., Middle East); where tobacco-consumption rates are alarmingly high and still increasing and where tobacco-consumption patterns are diverse.

## Early Smoking-Associated Molecular Changes in Lung Cancer Development

Premalignant lung lesions and NSCLCs in smokers share mutual molecular alterations that have been reviewed elsewhere (13). Studying these premalignant lesions in depth (e.g., by genome-wide profiling and sequencing) will undoubtedly improve our understanding of the early pathobiology of smoking-induced lung cancer development. By RNA-sequencing of a relatively small set of smoking-associated squamous premalignant and malignant lesions, the study by Ooi and colleagues pointed the role of aberrant *MYC* activation in the development and progression of squamous premalignant lesions following smoking (20). Ongoing recent studies have employed whole-exome sequencing to characterize recurrent driver mutations implicated in the pathogenesis of squamous dysplasias in smokers (21).

The concept of field cancerization was first observed and developed by Slaughter, describing sites of neoplasia and histologically adjacent normal-appearing tissue (22, 23). Additional studies probed the effects of smoking exposure on cytologically normal airway epithelial cells revealing that smoking perpetuates airway-wide molecular aberrations signifying a “field of injury” phenomenon that is very likely pertinent to lung oncogenesis (24–26). This phenomenon referred to as the “airway field of injury,” could provide insights into the early pathobiology of lung cancer development, and thus, clinical opportunities for early detection (e.g., in suspect smokers with indeterminate nodules) (12, 27). Various molecular alterations, such as mutations, copy number variations and DNA methylation, have been described in the smoking-associated airway epithelial field and are reviewed elsewhere (13, 28). A seminal study by Spira and colleagues underscored smoking-associated genome-wide expression changes in the cytologically normal airway (29). Notably, subsequent studies demonstrated the relevance and significance of gene expression changes in minimally invasive sites (e.g., mainstem bronchus) within the airway field of injury to early detection of lung cancer in smokers with suspicion of the disease (26, 27, 30, 31). Recent work revealed that the epithelial field of lung cancer-associated injury in ever smokers extends to the nose and has potential in early lung cancer detection (32). These reports provide strong support for the role of the airway field of injury in lung cancer development. Analysis of genome-wide DNA alterations in the cancerization or injury fields within the normal-appearing airway have been limited. The recent study by Jakubek and colleagues, utilizing genome-wide SNP arrays along with novel sensitive tools for analysis of subtle allelic imbalance, revealed loss-of-heterozygosity in driver oncogenes and tumor suppressors that are shared between the normal airway and NSCLC (33).

## Smoking Rates and Spectra in LMICs: Case of Lebanon

While non-communicable diseases, particularly cancer, pose a significant public health burden in high-income or upper middle-income countries, the effect is even more pronounced in low-income nations or LMICs. This is largely due to the relatively less developed healthcare systems in poorer economies (34). The bulk of the growing burden of cancer is sustained by LMIC (1, 35), where more than 20 million annual cancer diagnoses are projected for 2025 (1, 35). In high-income countries, cancer mortality rates reach 46% (36). Alarmingly, these rates are substantially higher in LMIC as the majority (~75%) of the nearly 7.5 million annual worldwide cancer deaths occur in LMIC (37, 38). These data strongly suggest a correlation between cancer survival rates and country income. Indeed, some studies have demonstrated disparate cancer survival rates between countries of different income groups or even within the same groups (39). Such geo-regional trends are also evident across the most frequently diagnosed type of malignancy worldwide, lung cancer, with 58% of the estimated 1.8 million new cases diagnosed in 2012 occurring in LMIC or developing countries (1).

**Figure 1:**
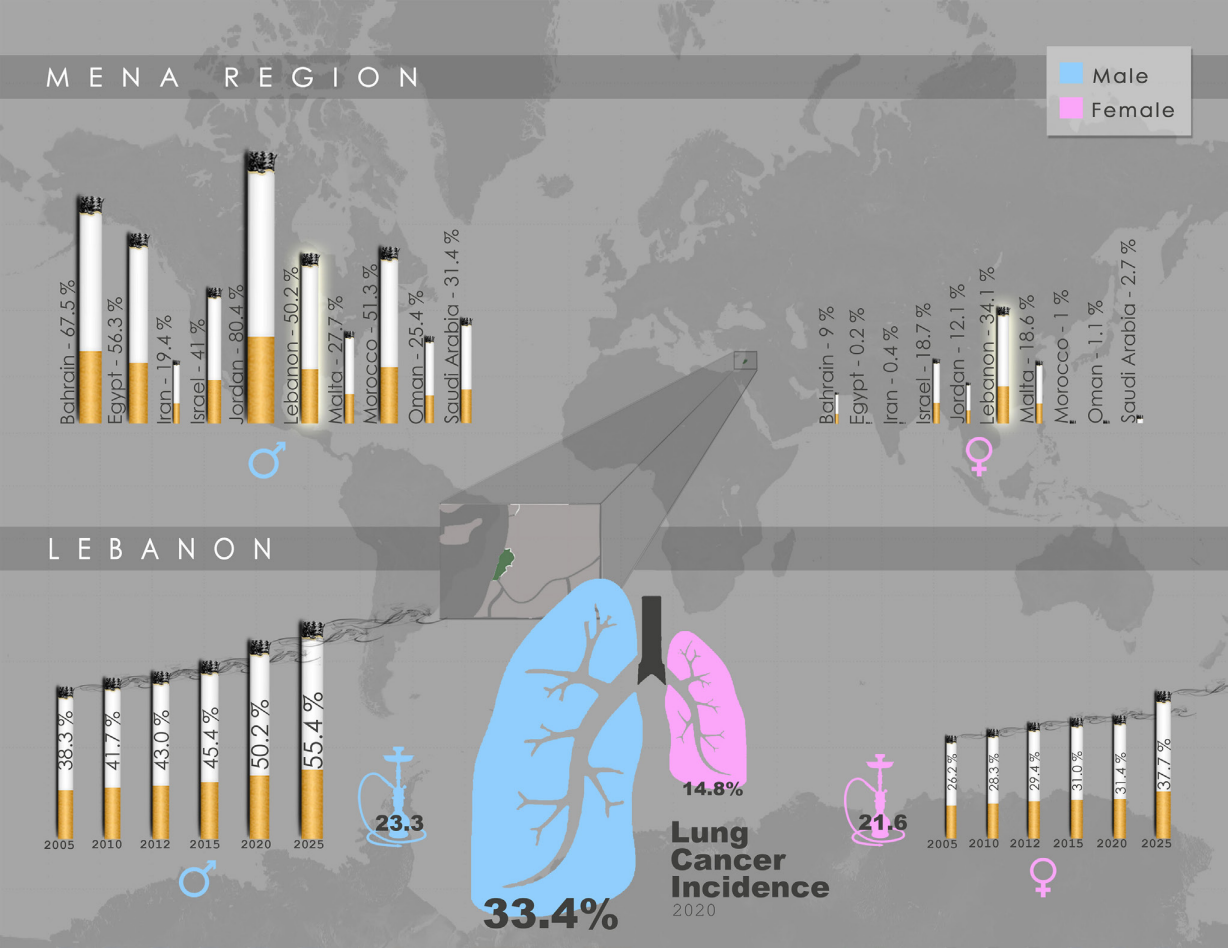
Gender-specific smoking prevalence in the Middle East and North Africa (MENA) region and Lebanon. Upper panel: Projected smoking prevalence of any tobacco product for 2020 in the MENA region, expressed as percentage of smokers aged ≥15 years across each gender. Lebanon exhibits the highest smoking prevalence among females, and the fourth highest among males. Lower panel: Trend in smoking prevalence of any tobacco product in Lebanon, expressed as gender-specific percentage of smokers aged ≥15 years, accompanied by water-pipe prevalence (2009), as well as lung cancer incidence projected for 2020. Data schematically presented here were obtained from WHO Global Health Observatory Data Repository by country, (45) and (46).

With smoking being directly associated with elevated risk of lung cancer, 80% of regions with the highest smoking rates among males (70–95%) are in developing countries (40). A report by the World Health Organization (WHO) revealed that the Middle East is one of only two regions in the world where cigarette consumption increased in the past few decades (41). In Lebanon, an LMIC within the Middle East, there is high prevalence of smoking among male adults with rates in the range of 50–60% (42) (Figure 1). Women in Lebanon, on the other hand, exhibit the highest age-adjusted smoking rates among females in middle eastern LMICs (42) (Figure 1). It is noteworthy that, even when compared to the US, a higher percentage of women were estimated to smoke or have smoked tobacco in Lebanon (43). Alarmingly, a recent report indicated that the per capita cigarette consumption rate in Lebanon rose by a striking 475% in the last few decades, the second highest increase globally, placing the country as the third worldwide in annual cigarette consumption per capita for both genders (44) (Figure 1). Taken together, these reports suggest that the high smoking prevalence rates in LMIC countries such as Lebanon will undoubtedly lead to a surge in lung cancer incidences, with the epidemic of NSCLC most likely yet to peak in these countries.

In addition to cigarette smoking, tobacco consumption in LMIC, specifically the Middle East and North Africa (MENA) region, also include variants such as water-pipe smoking (also known as hookah or narghile). This “tobacco smoking device” comprises either “tumbâk” (moistened raw tobacco) directly burned by charcoal, or “moassal” a fruit-flavored moist tobacco covered with aluminum and heated by charcoal (47). It is estimated that approximately 100 million people worldwide smoke tobacco in this manner using variations of the water-pipe (48–50). Water-pipes are used socially, frequently being shared among students, family and friends at home, or in dedicated cafes and bars (51, 52). A study conducted in the US revealed increased prevalence of water-pipe smoking among young adults aged between 18 and 24 years (7.8%) relative to the remaining adult population (1.5%) (53). In Lebanon, water-pipe smoking is more culturally accepted than smoking cigarettes (54), rendering the plausible prediction that this cultural acceptance can amplify the public health burden of water-pipe especially among the very young and adolescents. In addition, in Lebanon and other MENA LMICs, it is conceived, albeit wrongly, that water-pipe smoking is safer and a non-addictive alternative to cigarette smoking (55–57). Remarkably, females in Lebanon report similar epidemics in water-pipe smoking prevalence as in cigarette consumption, where they exhibit the highest rates in the MENA region (42, 58). On the other side, the Lebanese youth, aged between 13 and 15, also shows a striking water-pipe smoking prevalence of 59.5% (59). It is worthwhile to note that water-pipe smoking has become an increasing phenomenon worldwide, particularly among young adults (41, 49, 60). These worldwide emerging trends further accentuate the need to study the effects of water-pipe smoking on lung pathophysiology, particularly lung cancer. Earlier efforts have shed light on potential harmful pathophysiological effects of water-pipe smoking (51, 61–65). Although, cigarette smoking was shown to be associated with a significantly greater daily nicotine intake in comparison to water-pipe smoking (48), both induced similar cardiovascular effects, specifically both exhibited similar effects on systolic blood pressure (48, 66). A meta-analysis by Montazeri and colleagues probed a positive association between water-pipe smoking and lung cancer by performing a systematic search of articles indexed in main biomedical databases, published between 1962 and September 2014 (67). Nevertheless, despite its long history and current revival, studies on water-pipe smoking use and effects are generally limited, and additional harmful agents in water-pipe smoke need to be investigated. It is plausible that water-pipe smoking in MENA LMIC such as Lebanon may influence the genetic makeup of lung cancers in that region, a supposition that is yet to be determined. Our understanding of the effect of water-pipe smoking on the genome is almost negligible. A recent study by Walters and colleagues demonstrated that water-pipe smoking is associated with epigenetic changes in the small airway epithelium that translate to transcriptional modifications (68). Comprehensive genome-wide analysis of lung tumors diagnosed in exclusive water-pipe smokers may underscore genomic aberrations implicated in water-pipe smoking-mediated lung cancer pathogenesis, which may be distinct from pathways more typically influenced by cigarette smoking.

Although smoking remains the primary risk factor in respiratory diseases, up to three million premature deaths worldwide have been attributed to air pollution in 2012, 87% of which are in LMICs (69). Exposure to ambient air pollution is carcinogenic according to WHO’s International Agency for Research on Cancer, with particulate matter (PM), a mixture of solid and liquid particles of different sizes and chemical profiles suspended in the air, being the principal pollutant associated with lung cancer incidence (70). People residing in LMICs incur the highest burden of outdoor air pollution, in part due to poor solid waste management. Indeed, outdoor air pollution is a prominent lung cancer risk factor in Lebanon; where, an unprecedented peak in air pollution levels was recently reported in the wake of the 2015 waste management crisis (71). Unauthorized open air incineration sites have since emerged in the vicinity of highly populated areas in Lebanon as a consequence of the interruption of solid waste collection (71). Waste incineration is known to emit an amalgam of hazardous pollutants which include PM, greenhouse gases (CO_2_, CH_4_), sulfur dioxide (SO_2_), nitrogen oxides (NO*_x_*), volatile organic compounds, polycyclic aromatic hydrocarbons (PAHs), dioxins (polychlorinated dibenzo-dioxins and furans-PCDD/Fs), among many others (72–77). Of note, PAHs exposure is highly associated with cancer risk, particularly lung cancer (78–81). The burden imposed by PAHs is further accentuated by the emissions of diesel electric generators used in Lebanon due to inadequate power production capacity (82). Among the most carcinogenic high molecular weight PAH agents reported is benzo[a]pyrene (BaP), a principal constituent of tobacco smoke that is mainly incurred by inhalation (70).

A recent study probing levels of hazardous chemical profiles directly generated as result of waste incineration in Lebanon reported that daily averages of fine PM that can draw deep into the lung exceeded the 24-h WHO guidelines (83). Concentrations of metal markers of waste burning (lead, cadmium, titanium, arsenic, and others) increased by an alarming 98–1,448%, while those of 16 toxic PAHs more than doubled, with BaP reaching 2.3 times the control samples. Seventeen toxic PCDD/Fs were also remarkably elevated on a specific day with unparalleled peak of incineration and, thus, exposures. Based on these alarming figures, short-term (2-year) cancer risk from exposure to PCDD/Fs and PAHs was estimated to increase from about 1 in one million to 20 per million, and further up to 65 cases per million solely due to inhalation of PCDD/Fs (83). These estimates are likely to be conservative underestimate since they account for acute environmental exposure, through one mean of acquisition: inhalation.

## Perspective

Lung cancer is causally related to smoking and tobacco consumption. Thus, it cannot be emphasized enough that understanding the molecular underpinnings of smoking exposure and tobacco consumption will offer invaluable opportunities for enhancing our understanding of lung cancer pathogenesis, and subsequently, the early detection of this morbid malignancy. As discussed above, LMICs such as Lebanon exhibit high age-adjusted smoking rates and a wide spectrum of tobacco-consumption modes (e.g., water-pipes)—let alone additional exposure to carcinogenic environmental pollutants. Thus, it is reasonable to speculate that this “cocktail” of exposures, along with socioeconomic factors, may incur a high total somatic mutational burden due to exposure to various carcinogens from multiple sources: cigarettes, water-pipe, and/or ambient pollutants. Indeed, the alarming trends in smoking prevalence, waste incineration practices and air quality deterioration project an imminent epidemic peak in lung cancer in Lebanon, further imposing an additional burden on health care systems. This will undoubtedly inflict a heavy toll on the economy of the country and of other LMICs. The affected populations in Lebanon and other LMICs are undoubtedly worth investigating owing to their particular susceptibilities, genetic predisposition, and the diversity of mutagens they are exposed to. However, our understanding of the acquired genetic changes leading to lung cancer remains rudimentary in those developing countries. In addition, elucidating the molecular causal links between water-pipe smoking and lung disease can limit the fashionable use of this habit among people of all ages, particularly the youth, once data are properly translated into social awareness campaigns. A deeper understanding of the socioeconomic reasons leading to the alarmingly high tobacco-consumption rates among Lebanese women is also urgently warranted.

The complexity of early events underlying lung cancer pathogenesis is being countered by the development of affordable “omics”-based technology, therefore enabling faster identification of putative biomarkers for improved clinical management. Identification of molecular and genomic mechanisms underlying exposure to the multitude of lung carcinogens (e.g., cigarettes, water-pipe smoking, air pollutants) will allow us to define high-risk groups with better “resolution” and derive biomarkers for personalized (chemo)prevention in those regions. However, many of these state-of-the-art molecular and genomic tools remain restricted to specialized research laboratories and have yet to become the gold standard for lung cancer patients in LMIC, Lebanon being an example. In developed countries, there have been modest improvements in lung cancer awareness efforts, early detection, prevention, and new therapeutic strategies, thus, translating into improved survival rate. For instance, many western clinical studies have assessed the effectiveness of different bronchial gene-expression classifiers in improving the diagnostic performance of bronchoscopy in smokers with indeterminate pulmonary nodules (12, 26, 27). Molecular markers such as plasma protein levels (84), serum microRNA signatures (85, 86) and autoantibodies to lung tumor-associated antigens (87–89) were also demonstrated to be potential biomarkers for early detection of lung cancer. However, these modalities are still very investigational and have not been broadly adapted into clinical practice as the benefit is still not clear or proven. In sharp contrast, progress in clinical and precision-based cancer management in developing countries, facing an increase in smoking prevalence compared with the promising decline in developed countries, is still extremely lagging. Reduced social awareness of the significance of early detection and screening added to the financial barriers of poverty and stigma, underlie the relatively late cancer diagnosis in LMIC. Further, despite the urgent need for immediate and large-scale response in developing countries, only trivial resources are dedicated toward clinical cancer research and control (37, 40, 90).

A resurgence of attention to such topics in future funding priorities in LMIC and especially Lebanon will likely improve patient outcome by steering healthcare toward improved early diagnosis, as well as advancing a roadmap for prevention strategies of air pollution- or tobacco smoking-related diseases. On the long run, such approaches will not only act as social promoters for a healthier lifestyle, but they will also serve as catalysts for decreasing the health and economic burden associated with lung cancer, particularly in LMICs.

## Author Contributions

ZR, SN, and HK conceived the study. HC and AT reviewed the manuscript. ZR, SN, AS, and HK wrote the manuscript. All the authors approved the final manuscript.

## Conflict of Interest Statement

The authors declare that the research was conducted in the absence of any commercial or financial relationships that could be construed as a potential conflict of interest.
